# The Good, the Bad, and the Ugly: Neutrophils, Angiogenesis, and Cancer

**DOI:** 10.3390/cancers14030536

**Published:** 2022-01-21

**Authors:** Irem Ozel, Inga Duerig, Maksim Domnich, Stephan Lang, Ekaterina Pylaeva, Jadwiga Jablonska

**Affiliations:** Department of Otorhinolaryngology, University Hospital Essen, University Duisburg-Essen, 45147 Essen, Germany; irem.oezel@uk-essen.de (I.O.); inga.duerig@uk-essen.de (I.D.); maksim.domnich@uk-essen.de (M.D.); stephan.lang@uk-essen.de (S.L.); ekaterina.pylaeva@uk-essen.de (E.P.)

**Keywords:** tumor vasculature, neutrophils, cancer, tumor angiogenesis

## Abstract

**Simple Summary:**

Tumor angiogenesis is one of the most critical steps in the progression of cancer. Owing to its fundamental role in tumor growth and metastasis, tumor angiogenesis is accepted to be a limiting factor and considered a powerful therapeutic target. Neutrophils contribute to the tumor progression via multiple mechanisms, ranging from the direct support of tumor cell survival to the immunosuppression. A strong body of evidence suggests that neutrophils also play a prominent role in tumor angiogenesis. In this manuscript, we provide an up do date review of the pro-angiogenic functions of neutrophils, in the context of cancer, and discuss the possibility of therapeutically targeting the pro-angiogenic capacity of these cells in cancer patients.

**Abstract:**

Angiogenesis, the formation of new blood vessels from already existing vasculature, is tightly regulated by pro- and anti-angiogenic stimuli and occurs under both physiological and pathological conditions. Tumor angiogenesis is central for tumor development, and an “angiogenic switch” could be initiated by multiple immune cells, such as neutrophils. Tumor-associated neutrophils promote tumor angiogenesis by the release of both conventional and non-conventional pro-angiogenic factors. Therefore, neutrophil-mediated tumor angiogenesis should be taken into consideration in the design of novel anti-cancer therapy. This review recapitulates the complex role of neutrophils in tumor angiogenesis and summarizes neutrophil-derived pro-angiogenic factors and mechanisms regulating angiogenic activity of tumor-associated neutrophils. Moreover, it provides up-to-date information about neutrophil-targeting therapy, complementary to anti-angiogenic treatment.

## 1. Introduction

The functional vascular system, in complex a multicellular organism, is a very vital asset for survival. The network of blood vessels nurtures all the tissues by supplying oxygen and nutrients, as well as providing gateways for immune surveillance [1,2]. In the early development of the embryo, mesoderm-derived endothelial precursor cells (termed angioblasts) constituted vasculogenesis. In later stages of embryonic development, the tree-like structure of blood vessels were generated from this initial vasculature, in a process called “angiogenesis” [3]. Although the new vessel formation occurs mainly during the embryonic development, angiogenesis also takes place in the adult organism and plays a crucial role in physiological conditions, such as wound healing, placenta formation, and menstrual cycles [3]. Next to physiological angiogenesis, abnormal development of new blood vessels occurs in multiple pathological conditions, such as cancer. Tumor angiogenesis is essential for cancer progression because tumors cannot grow beyond 1 mm^3^ without sufficient blood supply [4]. Moreover, the spread of tumor cells is strongly dependent on functional vasculature [5]. Therefore, tumor cells release factors that modulate the activity of immune cells, including neutrophils, to support their pro-angiogenic activity. Herein, we recapitulate the contribution of neutrophils to the cancer angiogenesis and discuss their potential complementary role in the anti-angiogenic cancer immunotherapy.

## 2. Physiological Angiogenesis vs. Tumor Angiogenesis

Angiogenesis is the process of new blood vessel formation from already existing vasculature. The capillary of blood vessels consists of an endothelial cell (EC) monolayer, surrounded by the basal membrane and pericytes [1]. In the steady state, endothelial cells are non-proliferating and quiescent, and their activation is strongly coordinated by pro-angiogenic signals [6]. These pro-angiogenic signals occasionally appear, along with the demand for nutrients or oxygen in hypoxic and nutrient-deprived tissues [7]. In response to pro-angiogenic signals, two conventional mechanisms of angiogenesis are known: sprouting angiogenesis (SA) and intussusceptive angiogenesis (IA), which was discovered later [8]. SA contributes to vascular growth via the formation of sprouts from existing vessels, whereas IA is characterized by the splitting of already existing vessels into new ones [9]. IA is a relatively faster mechanism than SA, and it is mainly responsible for vascular remodeling [8]. SA and IA are reviewed in detail elsewhere [8,10].

In physiological conditions, angiogenesis is a strongly controlled process, which relies on complex interactions among endothelial cells, pericytes, vascular smooth muscle cells, and immune cells [1]. The initiation of angiogenesis is mediated by pro-angiogenic factors that are released from ischemic/hypoxic or nutrient-deprived tissues. Classical pro-angiogenic factors are: vascular endothelial growth (VEGFs), fibroblast growth (FGFs), platelet derived growth (PDGFs), angiopoietins (ANGs), hepatocyte growth (HGF), insulin-like growth (IGFs), tumor necrosis (TNF) [11], and interleukin 6 (IL-6) [12]. Those factors stimulate endothelial cells to form tip and stalk cells and support their migration towards angiogenic stimuli [12]. Tip cells are activated ECs that secrete the enzymes responsible for the degradation of extracellular matrix (ECM), such as matrix metalloproteinase-9 (MMP-9). Stalk cells show a highly proliferative profile and are responsible for the elongation of the sprout. These cells line the wall of sprouts and form the lumen that enable the blood flow [1]. 

Among many pathways, the VEGF–VEGFR axis is the most studied signaling pathway in angiogenesis [13]. In addition, numerous non-classical pro-angiogenic signals and auxiliary molecules have been identified, such as erythropoietin (EPO) [14], apelin [15], coagulation factor XIII (FXIII) [16], pro-inflammatory cytokines [17,18,19,20,21,22,23], or exosomes [24,25,26,27]. Anti-angiogenic factors, on the other hand, such as thrombospondin-1 [28], endostatin [29], tumstatin [30], interferon-α (IFN-α) [31], or IFN-β [32] limit the angiogenesis by maintaining the quiescent state of endothelial cells. The balance between pro- and anti-angiogenic factors is crucial for normal, functional vessel formation; therefore, its deregulation results in pathology and contributes to the progression of diseases, such as cancer.

Malignant cells, similar to normal cells, need oxygen and nutrients in order to proliferate. In its early stage, cancer grows in an avascular mode that is sufficient for cell proliferation and survival. At later stages, tumor cells become hypoxic and upregulate hypoxia-inducible factor-1 (HIF-1) [33], which supports the expression of pro-angiogenic molecules by binding to their promotors [34]. As the tumor grows and creates an uncontrolled proliferating cell mass, it becomes increasingly dependent on the vasculature. The importance of angiogenesis for cancer development was first proposed by Judah Folkman [4] and is now generally accepted [4,35,36,37,38,39,40]. With the increasing nutrient and oxygen demand, along with the hypoxic microenvironment of a newly growing tumor, expression of pro-angiogenic factors is accelerated. This shift of angiogenic balance is called “angiogenic switch”. 

Tumor vasculature can be established through conventional angiogenesis mechanisms (SA and IA), as well as via vascular mimicry or blood vessel co-option [10]. Two latter mechanisms (occasionally called non-angiogenic mechanisms) are limited to pathological conditions and do not depend on pro-angiogenic factors [13]. In vessel co-option, pre-existing tissue vessels are hijacked and incorporated into the tumor [13]. In vascular mimicry, tumor cells behave like ECs and form non-endothelial vessels [8]. Opposite to physiological angiogenesis, where the activity of ECs is tightly controlled, tumor angiogenesis is strongly accelerated through the constant pro-angiogenic signaling and results in the formation of disordered, partly non-functional vasculature. Tumor blood vessels consist of tumor endothelial cells (TECs), which are different from normal ECs by morphology, gene expression, and metabolism [41]. TECs are structurally abnormal, have weak intercellular junctions, and show increased proliferation and migration, in response to angiogenic signaling [41]. Moreover, it has been suggested that these cells are resistant to senescence [41]. 

Tumor cells autonomously produce pro-angiogenic signals; however, they could also prime immune cells, such as neutrophils, to further promote angiogenesis [41]. Tumor-associated inflammation also has an important role in tumor angiogenesis and progression [42]. In response to hypoxia, the expression of pro-inflammatory molecules is upregulated in TECs [43], leading to the recruitment and activation of inflammatory cells, such as macrophages and neutrophils, that contribute to the tumor angiogenesis. 

## 3. Neutrophils in Cancer

Neutrophils, as the most abundant leukocytes in human blood circulation, ranging between 50 and 70 percent (10–25 percent in mice) [44], are essential players in innate immunity and inflammatory responses. Nearly every day, new neutrophils (10^11^ cells in human) are produced in the bone marrow, in a process called granulopoiesis [45]. Neutrophils were initially believed to be terminally differentiated, homogenous, and short-lived cells; however, recent studies challenged this hypothesis and showed that neutrophils have high diversity and plasticity, with regard to functions [46,47,48,49,50,51,52,53,54,55]. Notably, growing tumors strongly affect the development and activity of neutrophils via a plethora of released growth factors and cytokines [46].

Tumor-Associated Neutrophils

The role of neutrophils in cancer is still controversial. High numbers of immature myeloid cells in the blood, including neutrophils (neutrophilia), is a frequently seen phenomenon in cancer patients, as well as in tumor-bearing mice [46,56,57]. Neutrophils efficiently infiltrate to tumors (tumor-associated neutrophils, TANs) and constitute a very important component of the tumor microenvironment (TME) [58,59]. In numerous pre-clinical and clinical studies, TANs have been shown to participate in malignant transformation, anti-tumoral immunity, and angiogenesis [56,60,61,62,63]. Moreover, an increased neutrophil-to-lymphocyte ratio (NLR) is now a well-recognized predictive factor for the progression of many cancer types [64,65,66,67,68]. 

**Figure 1:**
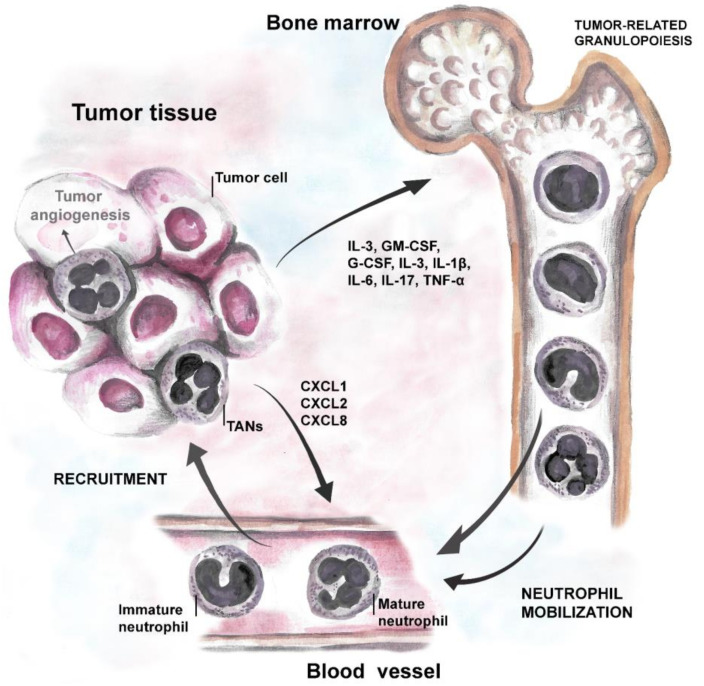
Tumor-neutrophil interactions. Tumor cells produce growth factors (IL-3, GM-CSF, and G-CSF) and inflammatory cytokines (IL-1β, IL-6, IL-17, and TNF) that induce neutrophil production and stimulate their survival. Chemoattractants specific for neutrophils, such as CXCL1, CXCL2 (ligands for CXCR2 on neutrophils), and CXCL8 (via CXCR1 on neutrophils) support their migration to the tumor site.

Strong body of evidence suggests that tumor cells produce growth factors (IL-3, GM-CSF, and G-CSF) or inflammatory cytokines (IL-1β, IL-6, IL-17 and TNF-α) to induce neutrophil production and support their survival [69,70]. Neutrophil-attracting chemokines, such as CXCL1, CXCL2, or CXCL8, are induced in hypoxic tumor tissue, through the activation of hypoxia-inducible factor 1 (HIF-1α or β) [46,57,71], and support neutrophil migration to the tumor site via CXCR1 and CXCR2 receptors [72]. Moreover, inflammatory cytokines stimulate G-CSF production in TME and further induce the generation of new neutrophils in bone marrow (cancer-related granulopoiesis) [73,74]. Neutrophils, in turn, contribute to cancer-related inflammation, thus promoting angiogenesis and tumor progression (Figure 1). 

Tumor-associated neutrophils can exert ambivalent functions, ranging from direct killing of tumor cells to promoting tumor angiogenesis, metastasis, and immunosuppression. Similar to tumor-associated macrophages (TAMs), the phenotype and activity of TANs show significant heterogeneity throughout cancer development [75,76,77,78,79]. At the early stage of cancer, neutrophils are mainly located at the periphery of the tumor and display anti-tumoral phenotype, while, at later stages of the disease, their pro-tumoral properties dominate and support further cancer progression [57,80,81,82,83,84,85]. The pro- or anti-tumoral phenotype of neutrophils in the primary tumor or metastatic site is highly dependent on the cytokine milieu [86]. The functional polarization of TANs, under the influence of TME, and their phenotypic characterization have been previously reviewed in detail elsewhere [46,49,52,56,76,87,88,89]. It has been shown that IFN signaling triggers the anti-tumor activity of TANs (N1), characterized by normal density and hyper-segmented nucleus [55,90]. These cells express high levels of TNF-α, CCL3, and ICAM-1; additionally, they show low arginase-1 expression and are cytotoxic towards cancer cells in the experimental setting [91]. TGF-β stimulation or defective type I interferon (IFN) signaling stimulates the pro-tumoral activity of TANs (N2), with low density and both a segmented/not segmented nucleus [75]. These neutrophils can exert immunosuppressive activity, with upregulated expression of CCL2, CCL3, CCL4, CCL8, CCL12, CCL17, CXCL1, CXCL2, IL8/CXCL8, and CXCL16 chemokines [91]. N2 neutrophils promote the genetic instability of cancer cells by producing reactive oxygen species (ROS) and the release of exosomes containing microRNAs that impair nuclear integrity [46]. Moreover, upon G-CSF and TGF-β stimulation, neutrophils express arginase-1 (ARG1), ROS, and nitric oxide (NO) that contribute to the suppression of T cells [75,92,93]. Importantly, it has also been shown that both circulating, and tumor-associated neutrophils could suppress T cell activation by using immune checkpoint molecules, such as PDL-1 (programmed cell death ligand 1) or VISTA (V-domain immunoglobulin suppressor of T cell activation) [94,95]. 

## 4. Neutrophils and Tumor Angiogenesis

Neutrophils have been shown to modify their angiogenic capacity under the influence of tumor-derived factors, even before arriving at the TME [96]. Bone marrow neutrophils isolated from naive mice have been reported to suppress tumor growth, while their counterparts from tumor-bearing mice promoted tumor growth [96]. It is generally accepted that the pro-angiogenic properties of tumor-associated neutrophils support cancer progression. The involvement of TANs in tumor angiogenesis was already proposed, in 2006 by Nozawa et al., in a murine pancreatic islet carcinogenesis model. In this study, neutrophils were identified as an important source of MMP-9, and their functional involvement in tumor angiogenesis was demonstrated by the suppression of tumor angiogenic switch after depletion with the anti-Gr-1 antibody [97]. Following this study, the pro-angiogenic activities of neutrophils have been suggested in other cancer types, such as hepatocellular carcinoma, gastric cancer, melanoma, and fibrosarcoma [32,98,99,100]. In the liver tumorigenesis model, enhanced hypoxia and inflammation in TME have been shown to support the recruitment of neutrophils and promote tumor angiogenesis [98]. Nagaoka et al. demonstrated that IL-17-induced neutrophil infiltration, in a progressive tumor model of gastric cancer, increases tumor angiogenesis and supports tumor persistence [101]. Furthermore, the important role of neutrophil clustering in the vascularization of nasal cavity cancer (nasal inverted papilloma, NIP) was demonstrated recently [102]. MMP-9 and HIF-1α expressing neutrophils were shown to be the main tumor-infiltrating cells that promoted angiogenesis and significantly contributed to the NIP pathogenesis [102]. 

In agreement with the essential role of neutrophils in tumor angiogenesis, reduced tumor vascularization and growth have been demonstrated after antibody-mediated neutrophil depletion (using anti-Gr-1 or anti-Ly6G antibody) or inhibition of neutrophil infiltration into tumor tissue (e.g., anti-vascular cell adhesion molecule-1 and anti-VCAM-1) in several studies [32,97,100]. Adoptive transfer of anti-angiogenic neutrophils has also been shown to limit tumor angiogenesis in the murine model of melanoma [103,104,105], while the transfer of pro-angiogenic neutrophils supports tumor vascularization in mice [32,105]. 

Neutrophils have been recently shown to contribute to tumor vascularization, also through non-angiogenic mechanisms, vessel co-option, and vascular mimicry [106,107]. High neutrophil expression of *LOXL4* in colorectal cancer liver metastases (mainly via vessel co-option) has been proposed to be the key factor responsible for the resistance against anti-angiogenic therapy [108]. In a very recent study, vascular mimicry (VM) structures (that consisted of cancer-associated fibroblasts (CAFs) and tumor cells) were shown to provide channels for neutrophil infiltration in lung cancer [107]. Such VM structures induced the pro-angiogenic (N2) polarization of infiltrating neutrophils and promoted their arginase, CCL2, CXCR4, and MMP-9 expression and, thus, evade anti-angiogenic therapy [107].

### 4.1. Pro-Angiogenic Switch of Neutrophils in Cancer

**Figure 2:**
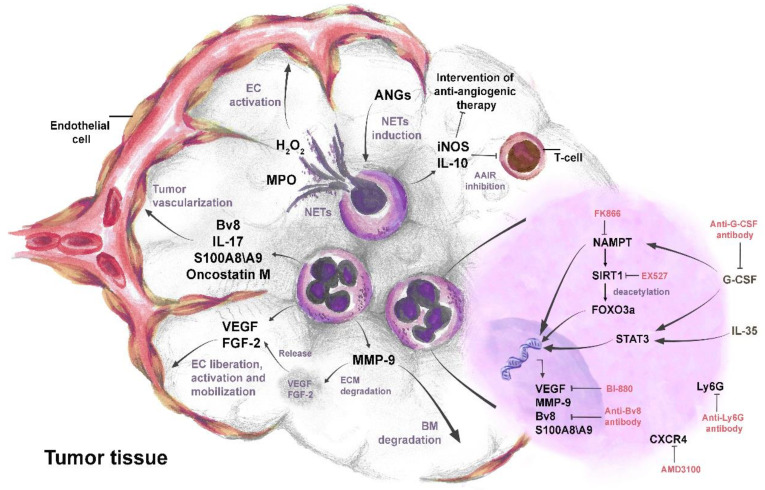
The complex role of neutrophils in tumor angiogenesis. IL-35- and G-CSF-mediated activation of STAT3 and NAMPT pathways leads to the up-regulation and secretion of pro-angiogenic factors VEGF, FGF-2, oncostatin M, IL-17, Bv8, MMP-9, and S100A8/S100A9 in neutrophils. The activated FOXO3a transcription factor also contributes to the production of pro-angiogenic factors. Pro-angiogenic factors released from neutrophils directly promote liberation, proliferation, and mobilization of endothelial cells (ECs) and induce tumor angiogenesis. Neutrophil MMP-9 mediates the degradation of the basal membrane (BM) and extracellular matrix (ECM). Degradation of ECM by MMP-9 leads to the release of sequestered VEGF and FGF-2 in ECM. Angiopoietins (ANGs) induce neutrophil extracellular trap (NET) formation in neutrophils. MPO in NETs increases H_2_O_2_ and stimulates proliferation and mobility of ECs. Neutrophils secrete IL-10 and iNOS to suppress anti-angiogenic adaptive immune response (AAIR) and later sustain tumor escape from anti-angiogenic therapy. Anti-Ly6G, anti-G-CSF, and anti-Bv8 antibodies inhibit neutrophil mediated tumor angiogenesis. Targeting NAMPT, SIRT1, VEGF, and CXCR4 in neutrophils, using molecular inhibitors such as FK866, EX527, BI-880, and AMD3100, respectively, reduces their pro-angiogenic activity.

Neutrophils support the pro-angiogenic switch during cancer development [97] and are significant producers of pro-angiogenic factors in TME [109,110]. Pro-angiogenic factors that are released by neutrophils, such as VEGF, Bv8, MMP-9, and S100A8/S100A9, directly induce tumor angiogenesis via the activation of endothelial cell proliferation [32,50,97,111]. Human neutrophils have been shown to carry an intracellular pool of VEGF and mediate its rapid secretion, through the degranulation upon stimulation with phorbol-12-myristate 13-acetate (PMA) and TNFα [112] (Figure 2).

In addition to their direct pro-angiogenic function, neutrophils can also activate pro-angiogenic functions of other immune cells and, thus, indirectly contribute to angiogenesis. It was previously demonstrated that human neutrophils can induce regulatory-like phenotypes of T cells and support their expression of IL-10, IL-17, and VEGF to promote vessel growth in pregnancy [113]. Possibly similar mechanisms could take place in the tumor microenvironment. Neutrophils could also affect anti-tumoral adaptive immune responses to support tumor angiogenesis [114]. In one study, Zou et al. identified pro-angiogenic immunosuppressive N2 neutrophils by producing iNOS inhibited T cell activation and suppressed anti-tumor adaptive immune responses, thus promoting tumor cell survival and proliferation [114]. 

Cancer metastasis constitute a major challenge in effective cancer therapy. Pro-angiogenic neutrophils have also been shown as significant supporters of tumor cell detachment and cancer metastasis [115,116]. In an animal model of breast cancer, JUNB deficiency was shown to increase neutrophil infiltration in the metastatic lung. Such neutrophils released elevated amounts of MMP-9 and BV8, leading to enhanced lung metastasis [117]. 

Importantly, neutrophils can also interfere with anti-angiogenic therapies. It was shown that the infiltration of CD11b^+^ Gr1^+^ myeloid cells (neutrophils) into tumor significantly reduced the efficacy of anti-VEGF antibody therapy [118]. Moreover, by secreting immunosuppressive cytokine IL-10 and hampering of T cell proliferation, neutrophils were shown to sustain tumor escape from anti-angiogenic therapy [119]. 

### 4.2. Neutrophil-Derived Factors That Support Tumor Angiogenesis

Neutrophils have been shown to support tumor angiogenesis via the release of numerous pro-angiogenic factors, such as VEGF, FGF-2, oncostatin M, IL-17, MMP-9, Bv8, S100A8/9 alarmins, STAT3, and NETs. Identification of other possible pro-angiogenic factors could support further approaches in successful cancer therapy. 

#### 4.2.1. VEGF and FGF-2

VEGF is a major pro-angiogenic factor, and VEGF signaling plays a central role in both physiological and pathological angiogenesis. It has been shown that human neutrophils carry an intracellular pool of VEGF and release it during the process of degranulation, upon stimulation with PMA, fMet-Leu-Phe (fMLP), and TNF-α [112]. Interestingly, neutrophils were found to be the main source of the circulating VEGF in the majority of patients with metastatic breast cancer or anal carcinoma [120]. Another study has reported that circulating neutrophils release higher amounts of VEGF in patients with oral cavity cancer, compared to healthy donor counterparts [121]. Furthermore, it has been shown that neutrophil depletion is linked to the inhibition of angiogenesis because of the reduced release of pre-formed neutrophil VEGF stores [122]. Importantly, elevated infiltration of tumor tissue by CD11b^+^Gr^+^ neutrophils, characterized by the increased expression of *VEGF*, was demonstrated to contribute to tumor angiogenesis in a mouse model of fibrosarcoma and melanoma [32]. 

Surprisingly, VEGF was also shown to indirectly affect neutrophil recruitment. It was reported that IL-8 expression by endothelial cells after VEGF stimulation induced neutrophil migration in vitro [123]. Moreover, VEGF production by neutrophils might promote the further recruitment of neutrophils, in a positive feedback loop, to establish pro-angiogenic signaling inside the tumor and support tumor angiogenesis.

Additionally, neutrophils have been shown to produce and/or contribute to the release of FGF-2. The direct FGF-2 expression of tumor-infiltrating neutrophils was demonstrated in the mouse model of hepatic metastases from gastrointestinal tumors [116]. In this study, neutrophils from hepatic metastases were shown to abundantly express FGF-2 mRNA and promote angiogenesis. Furthermore, neutrophil activity stimulates the release and bioavailability of sequestered FGF-2 in the extracellular matrix [124,125].

#### 4.2.2. Oncostatin M and IL-17

Oncostatin M is a cytokine produced by T cells, monocytes, and neutrophils. In early studies, oncostatin M has been shown to inhibit cancer cell proliferation and tumor growth [126,127]. However, neutrophil-secreted oncostatin M has been suggested to contribute to angiogenesis and invasiveness of breast cancer [115]. When co-cultured with breast cancer cells in vitro, human blood neutrophils have been shown to express oncostatin M [115]. Furthermore, both the cell–cell contact and GM-CSF production of breast cancer cells were demonstrated to be necessary for the oncostatin M release from neutrophils. Importantly, GM-CSF-dependent production of oncostatin M by neutrophils has been shown to promote the invasiveness and VEGF expression of MDA-MB-231 and T47D human breast cancer cells [115]. 

Neutrophil-derived IL-17 has also been suggested to induce tumor angiogenesis [128]. Numasaki et al. demonstrated that IL-17 enhances the angiogenic activity and in vivo growth of non-small cell lung cancer (NSCLC) by promoting the expression of angiogenic chemokines (CXCL1, CXCL5, CXCL6, and CXCL8) [128]. Interestingly, tumor infiltrating T cells and neutrophils were identified as the major source of IL-17 in NSCLC tissue [128]. 

#### 4.2.3. MMP-9

MMPs are reported to be highly expressed in various cancer types and are commonly associated with the tumor progression [129,130,131]. MMP-9 is a gelatinase that mediates the remodeling of the extracellular membrane, and it has been demonstrated to play an important role in tumor angiogenesis by triggering the angiogenic switch [132]. Importantly, tumor-associated neutrophils were reported to have significantly more abundant MMP-9, readily available for rapid release, than tumor-associated macrophages [133]. Neutrophil-produced MMP-9 mediates degradation of the basal membrane and leads to the liberation and activation of ECs. The degradation of the basal membrane, by MMP-9, indirectly leads to the release of pro-angiogenic factors VEGF and FGF-2, stored in extracellular matrix [97,116,125,134]. VEGF, sequestered in the tumor extracellular matrix, after being released by MMP-9, increases further neutrophilic expression of MMP-9 in a positive feedback fashion [135]. VEGF-A was also demonstrated as a chemoattractant for MMP-9-expressing neutrophils [136]. Moreover, increased levels of VEGF-A in hypoxic regions recruit a distinct subpopulation of pro-angiogenic neutrophils that are characterized by the expression profile of CD49d^+^VEGFR1^hi^CXCR4^hi^ [137]. Neutrophil MMP-9 also increases VEGF-A bioavailability and bioactivity and contributes to the inflammation-induced lymphangiogenesis [138]. Li et al. recently identified high levels of IL-17^+^ neutrophils as the major source of MMP-9 in the invasive margin of gastric cancer [139]. In this study, IL-17 producing neutrophils were shown to promote the pro-angiogenic activity of cancer cells, both in vivo and in vitro. Moreover, such neutrophils stimulated cancer cells to produce neutrophil chemoattractants, which, in turn, supported further neutrophil recruitment [139]. The pro-angiogenic role of MMP-9-secreting neutrophils in tumor vascularization was also validated in other types of cancer. In primary tumors formed by highly disseminating variants of human fibrosarcoma and prostate carcinoma, an elevated accumulation of MMP-9-positive neutrophils was demonstrated. The inhibition of neutrophil recruitment into such tumors, by IL-8 neutralization, diminished tumor angiogenesis [140].

#### 4.2.4. Bv8 and S100A8/9 Alarmins

Neutrophil-derived Bv8 and S100 proteins (S100A8 and S100A9) increase tumor proliferation and angiogenesis by supporting neutrophil mobilization, EC activation, and proliferation [46,141,142,143]. Tumor-associated neutrophils have been shown to produce Bv8 and S100A8 in a mouse melanoma model [105]. Bv8 has been suggested as a modulator of myeloid-cell-dependent tumor angiogenesis, and neutrophils have been reported to produce Bv8 robustly, after stimulation with G-CSF or GM-CSF [142,144]. Recently, Itatani et al. demonstrated that tumor-infiltrating neutrophils strongly expressed Bv8 in a mouse model of colorectal cancer. Importantly, in the same study, significantly higher plasma levels of Bv8 were observed in CRC patients, compared to healthy donors. Moreover, high plasma levels of Bv8 positively correlated with a bad prognosis and decreased overall survival of such patients [145]. In another study, treatment with anti-Bv8 antibodies reduced the number of angiogenic islets in transgenic mouse model of multistage pancreatic β-cell tumorigenesis [111]. Moreover, such anti-Bv8 treatment inhibited the recruitment of CD11b^+^Gr^+^ neutrophils to the neoplastic lesions [111,118,146]. Apart from direct stimulation of tumor angiogenesis, neutrophil-derived Bv8 also plays a role in tumor resistance to anti-VEGF therapy (see chapter 7) [145]. Increased levels of plasma G-CSF, after anti-VEGF therapy of mice bearing VEGF-resistant colorectal tumors, were shown to promote neutrophil infiltration into tumor stroma and stimulate angiogenesis via elevated neutrophil Bv8 expression [145]. This study demonstrates that blocking neutrophil-derived Bv8 increases the efficacy of anti-VEGF antibody therapy in colorectal cancer [145]. 

#### 4.2.5. STAT3

STAT3 is transcription factor that is involved in cell proliferation, survival, and angiogenesis [147]. STAT3 has been reported to be constitutively activated in both tumor and immune cells in the tumor microenvironment [147,148,149]. Neutrophil STAT3 activity (triggered via G-CSF/G-CSFR pathway) is important for their production, survival, and mobilization from the bone marrow [150,151,152]. STAT3 signaling in mice has been shown to have a regulatory role in neutrophil pro-angiogenic activity by controlling the expression of *VEGFA*, *FGF-2*, and *MMP-9* genes [153]. Importantly, it has also been demonstrated that neutrophils isolated from tumors have activated STAT3 and induce angiogenesis in vitro [153]. STAT3 signaling in neutrophils was suggested to activate neutrophil pro-angiogenic functions, even before they entered the TME [96]. Stimulation by G-CSF and IL-6 was shown to increase Stat3 expression, which then led to the upregulation of *MMP-9* and *Bv8* genes in bone marrow neutrophils [96]. Mechanistically, G-CSF-mediated STAT3 activation induces binding of phospho-STAT3 to the Bv8 promotor and elevates the expression of Bv8 [154]. The inhibition of Stat3, by type I IFN signaling, impairs VEGF and MMP-9 production by neutrophils and suppresses tumor angiogenesis [32]. 

### 4.3. The Role of Neutrophil Extracellular Traps in Angiogenesis 

Neutrophils contribute to inflammatory angiogenesis, also by neutrophil extracellular trap (NET) formation. Myeloperoxidase that is attached to such NETs increases H_2_O_2_ in ECM and leads to the activation of NFκB-mediated inflammatory signaling in ECs via TLR4. This initiates proliferation and mobility of endothelial cells and, as a result, tumor angiogenesis [155]. It has previously been shown that cancer cells are able to induce NET formation by neutrophils and, thus, to promote tumor angiogenesis [156]. Angiopoietins (ANG-1 and ANG-2) also were shown to induce NETs formation and contribute to pro-inflammatory and pro-angiogenic activities of neutrophils [157]. Angiopoietin-induced NETosis of human neutrophils promoted neutrophil adhesion to HUVECs and stimulated their proliferation [157]. 

## 5. Mechanisms Regulating Pro- and Anti-Angiogenic Activity of Neutrophils in Cancer 

Neutrophil angiogenic activity can be modulated by multiple tumor-released factors. Many signaling pathways have been suggested to promote neutrophil pro-angiogenic functions. Type I interferon signaling is one of the most prominent pathways that has been shown to strongly influence angiogenic potential of neutrophils. Owing to their ability to promote anti-tumoral (N1) neutrophil differentiation, IFNs inhibit neutrophil expression of pro-angiogenic factors. It has been demonstrated that in vivo transfection of IFN-β-expressing vector downregulated *MMP-9* and *Bv8* expression in neutrophils isolated from the bone marrow of tumor-bearing mice. This process was shown to be STAT3-dependent [96]. Another study showed that the deficiency of type I IFN supports pro-angiogenic switch of tumor-associated neutrophils and enhances tumor angiogenesis and growth [32]. Tumors from IFN-β^−/−^ mice show increased infiltration with pro-angiogenic neutrophils, characterized by the elevated levels of STAT3, MMP-9, and VEGF [32]. Transfer of such pro-angiogenic neutrophils into WT mice significantly elevated tumor growth. Importantly, the treatment of IFN-β^−/−^ neutrophils with rmIFN-β reduced their pro-angiogenic activity [32]. Similarly, IFN receptor-deficient mice (Ifnar^−/−^) showed elevated tumor angiogenesis and neutrophil infiltration in the tumor tissue [105]. Neutrophils from such mice significantly up-regulated their expression of *VEGF*, *MMP-9*, *Bv8*, and *S100A8* [105].

It has been demonstrated that the pro-angiogenic properties of neutrophils are stimulated by the activation of nicotinamide phosphoribosyl transferase (NAMPT) signaling pathway, downstream of G-CSF receptor [104]. It was shown that NAMPT expression in pro-angiogenic neutrophils is significantly upregulated and that the inhibition of NAMPT by the small molecule inhibitor FK866 impaired the pro-angiogenic phenotype of such tumor-associated neutrophils. Transfer of such neutrophils into tumor-bearing mice reduced tumor angiogenesis and growth [103,104]. 

Recently, another mechanism was shown to regulate the angiogenic potential of tumor-associated neutrophils. Bordbari et al. demonstrated that the pro-angiogenic capacity of neutrophils is strictly regulated by the post-translational modification of FOXO3a transcription factor that controls transcription of pro-angiogenic genes [105]. SIRT1-mediated deacetylation of FOXO3a was shown to activate this factor, while the phosphorylation, in the absence of SIRT1, led to cytoplasmic transfer and deactivation of FOXO3a [105]. Activated FOXO3a has been demonstrated to induce the transcription of pro-angiogenic molecules like VEGF, MMP-9, BV8, and S100A8 in TANs [105].

The CXCR2 receptor, on neutrophils, is the main regulator of neutrophil chemotaxis and function. Elevated expression of CXCR2 ligands (CXCL1, 2, 3, 5, 6, 7, and 8) is observed in various tumor types, such as lung cancer [158,159,160], hepatocellular carcinoma [161], renal cell carcinoma [162], and melanoma [72]. These chemokines can be directly secreted by cancer cells, endothelial cells, and cancer-associated fibroblasts, as well as by immune cells, including neutrophils [72]. High expression of CXCR2 ligands has been correlated with tumor aggressiveness in several types of tumors [163]. Moreover, CXCR2 depletion has been shown to have a negative effect on tumor growth and angiogenesis in a mouse model of lung cancer [164] and melanoma [72]. However, Timaxian et al. showed that the loss of CXCR2 in a murine breast cancer model resulted in increased growth of the primary tumor and lung metastasis. In that study, increased infiltration of tumors by pro-angiogenic TANs was observed [165]. Possibly, other neutrophil-attracting chemokines (such as CXCR4) compensated for the loss of CXCR2 in this experimental setting and attracted pro-angiogenic neutrophils into the tumor tissue. Nonetheless, this phenomenon must be further investigated. 

One such chemokine could be CXCL12 (a ligand for CXCR4), which was described to be overexpressed in melanoma [32] and other tumors [166]. It has been previously shown that CXCL12, in co-operation with IL-8, promotes angiogenesis in pancreatic cancer; therefore, CXCR4 could be a potential anti-angiogenic therapeutic target [167]. In a recent study, Tulotta et al. demonstrated the role of CXCR4/CXCL12 signaling in neutrophil-tumor cell interactions [168]. Importantly, pro-angiogenic neutrophils have been shown to express high CXCR4 in different studies [32,137]. 

Recently, IL-35 was shown to promote neutrophil infiltration and pro-angiogenic activity of neutrophils in TME. By inducing the polarization of neutrophils towards pro-angiogenic N2 phenotype, IL-35 supports the release of MMP-9 and Bv8 from neutrophils [114]. Additionally, in the same study, IL-35 has also been demonstrated to induce the activation of STAT3 and ERK pathway in neutrophils to upregulate their iNOS production, in order to suppress anti-angiogenic function of T cells [114].

Not only can cytokines or growth factors can modulate angiogenic activity of neutrophils. One isoform of plasma membrane vacuolar ATPase (a2V) was found to be secreted in several cancer types and suggested as a factor that mediates neutrophil recruitment to tumors [169]. Neutrophils treated with cancer-associated a2V showed prolonged survival [170] and elevated pro-angiogenic activity, with elevated expression of MMP-9 and VEGF [171]. 

Besides tumor cells, cancer-associated fibroblasts (CAFs) can also induce neutrophil pro-angiogenic activity and support tumor angiogenesis. CAFs in the lung cancer have been shown to express high levels of neutrophil chemoattractants, such as IL-6, IL-8, and CXCL1, and further support neutrophil recruitment by increasing their surface expression of VCAM and ICAM2 [107]. These CAFs significantly promote the infiltration of arginase+ N2 neutrophils and stimulate their expression of CXCL2, CXCR4, and MMP-9 [107]. Furthermore, it has been shown that CAFs mediate the activation of TGF-β/Smad3 signaling in tumor cells to induce IL-8 secretion and promote MPO^+^ neutrophil migration in lung cancer [172]. These MPO^+^ neutrophils have been reported to contribute to the tumor progression, through the support of angiogenesis. Additionally, by releasing MMP-9, these neutrophils were suggested to promote further activation of TGFβ in tumor microenvironment [172]. 

The effect of natural killer cells (NK cells) on angiogenic properties of neutrophils has been indicated [173]. By producing IFNγ and GM-CSF, NK cells have been shown to activate neutrophils and induce their surface expression of CD11b, CD62L, and CD64 [173]. After the activation by NK-derived factors, such neutrophils supported tumor angiogenesis by releasing VEGF and MMP-9. Moreover, they upregulated ARG1 expression, resulting in tumor growth [173], possibly by suppressing anti-tumoral T cell functions. On the other hand, Ogura et al. demonstrated that NK-derived IFNγ is able to inhibit VEGF expression of neutrophil [174], once again showing the complexity of the cell–cell interactions in tumor angiogenesis. 

## 6. The Role of Neutrophils during Anti-Angiogenic Therapy and Possible Therapeutic Approaches

The very first studies on anti-angiogenic therapies have focused on blocking VEGF/ VEGFR signaling with specific monoclonal antibodies [175]. Bevacizumab (VEGF-A mAb) or ramucirumab (VEGFR2 mAb) are the foremost Food and Drug Administration (FDA) approved drugs in the treatment of angiogenesis in cancer. However, these drugs reached a very limited success in the clinical trials, due to the tumor resistance [175], and it has been suggested that neutrophils play a significant role in this phenomenon [176]. Therefore, a growing body of evidence suggests therapeutic approaches, aiming at the inhibition of neutrophil pro-angiogenic role (summarized in Table 1).

The effect of neutrophils on the resistance to anti-VEGF therapy was demonstrated by Jung et al., who showed that the increased neutrophil infiltration after anti-VEGFR2 administration promotes tumor angiogenesis and decreases the efficacy of the therapy [119]. In that study, it has been shown that Ly6C^lo^ monocytes present in TME facilitate the recruitment of Ly6G^+^ neutrophils after anti-VEGFR2 therapy via the release of CXCL5. Interestingly, combining a neutrophil-targeting treatment with an anti-angiogenic therapy by the administration of anti-Ly6G antibody has been shown to reverse this effect, limit angiogenesis, and to support anti-VEGFR2 therapy [119]. The same research group demonstrated in another study that the anti-angiogenic treatment increases the expression of CXCL12 in tumors and leads to the recruitment of CXCR4^+^ pro-angiogenic neutrophils [177]. The blockade of CXCR4, by an FDA-approved agent AMD3100, inhibited Ly6G^+^ neutrophil infiltration into the tumor and improved the efficacy of anti-VEGF therapy [177]. 

Enhanced tumor inflammation, upon anti-VEGF therapy, was suggested as another reason for neutrophil infiltration into metastatic tumors of colorectal cancer (CRC) patients. The presence of CD177^+^ tumor-infiltrating neutrophils was demonstrated by immunostainings of TMAs, derived from colorectal metastasis samples [176]. The combination of traditional anti-angiogenic therapy (bevacizumab) and novel bi-specific VEGF/Ang2 blocking nanobody BI-880 diminished neutrophil infiltration and efficiently reduced angiogenesis, tumor growth, and hypoxia [176]. 

It has been shown that mice bearing VEGF-resistant colorectal tumors have elevated levels of plasma G-CSF, which leads to strong mobilization of neutrophils, their tumor infiltration, and induction of Bv8 expression that supports tumor angiogenesis [145]. Anti-VEGF therapy, in combination with anti-G-CSF or anti-Bv8/PROK2 antibodies, was demonstrated to efficiently suppress tumor growth [145]. 

Recently, a novel tool to manipulate pro-angiogenic capacity of TANs was suggested. The molecular inhibitor of NAMPT (FK866) was shown to repolarize neutrophils into an anti-angiogenic state [103,104]. Transfer of such anti-angiogenic TANs into tumor-bearing mice significantly impaired tumor growth. Moreover, histological examinations proved the significant suppression of angiogenesis in such tumors [103]. Furthermore, targeting neutrophil deacetylase SIRT1, which is a downstream target of NAMPT and, at the same time, an activator of pro-angiogenic transcription factor FOXO3a, was shown to suppress the pro-angiogenic activity of TANs [105].

## 7. Future Perspectives

Neutrophils contribute to tumor angiogenesis by direct secretion of pro-angiogenic molecules and the stimulation of endothelial cell proliferation. Furthermore, neutrophil recruitment and pro-angiogenic activity constitute a handicap in effective anti-angiogenic therapy. Standard anti-VEGF treatment showed, so far, moderate efficacy due to tumor resistance. Moreover, the growing body of evidence suggests that anti-VEGF therapy attracts neutrophils, induces their pro-angiogenic properties and leads to tumor therapy resistance. Therefore, going beyond the ordinary and designing therapeutic approaches simultaneously targeting neutrophils and tumor angiogenesis are necessary to provide a successful cancer treatment. In fact, combining anti-angiogenic therapy and immunotherapy has already been demonstrated as a beneficial strategy in cancer treatment [178,179]. Inhibition of neutrophil infiltration into tumors hold great promise to be the complementary to the traditional anti-angiogenic cancer therapy. G-CSF, GM-CSF, CXCR2, and CXCR4 are the most promising targets to control neutrophil migration. A growing body of evidence has been validated that inhibiting or manipulating neutrophil pro-angiogenic pathways, using molecular inhibitors or antibodies, in combination with anti-VEGF therapy, improves the outcome of anti-angiogenic therapy. In order to provide efficient delivery of anti-angiogenic molecules to neutrophils, another way of delivery should be re-considered. One such possibility could be nanoparticles, as these structures has been already demonstrated in various studies to effectively deliver their cargo, such as siRNA or proteins, into target cells [180]. Human and mouse neutrophils were shown to be successfully transfected with siRNA by using CD177-mediated nanoparticles [181]. Such technology could be optimized for the delivery of siRNA or proteins targeting the neutrophil release of pro-angiogenic factors in TME.

## 8. Conclusions

Neutrophils are one of the major players in regulating cancer growth from the early stages. Neutrophil-mediated tumor angiogenesis and metastasis handicap achieving a success in the treatment of many types of cancer. Nevertheless, targeting both angiogenesis and neutrophil functions in cancer shows a lot of potential and could take cancer immunotherapy a step further. 

## Figures and Tables

**Table 1:** Complementary neutrophil-targeting, anti-angiogenic therapy approaches.

Therapy	Compound	Target	Reference
Anti-Ly6G	Monoclonal antibody	Neutrophil recruitment	[119]
AMD3100	CXCR4 inhibitor	Neutrophil recruitment	[177]
BI-880	bi-specific VEGF/Ang2 blocking nanobody	Neutrophil recruitment	[176]
Anti-G-CSF	Monoclonal antibody	Neutrophil recruitment/production	[145]
Anti-Bv8/PROK2	Monoclonal antibody	Neutrophil Bv8	[145]
FK866	Molecular inhibitor	Neutrophil NAMPT	[103]
EX527	Molecular inhibitor	Neutrophil SIRT1	[105]
